# Dr. George Watt

**Published:** 1887-09

**Authors:** 


### Dr. George Watt.

We with thousands of others have occasion for thankfulness and even rejoicing in the fact, that our friend and brother, Dr. Watt, finds himself to-day in better health than for several years.

His health and strength have been so feeble that it has been a marvel to his friends that he could accomplish so much in journalistic work and do it so well; it has called forth the oft repeated remark, "It is a notable case of giant will-power dominating exceedingly adverse circumstances and conditions."

In a letter just received from him he remarks "I'm a boy again." We hope and pray for many years of good health and usefulness. It has always been a matter of wonder to us why our brother suffered so much. Perhaps we will know sometime.

